# *Salvia officinalis*–Hydroxyapatite Nanocomposites with Antibacterial Properties

**DOI:** 10.3390/polym15234484

**Published:** 2023-11-22

**Authors:** Steluta Carmen Ciobanu, Daniela Predoi, Mariana Carmen Chifiriuc, Simona Liliana Iconaru, Mihai Valentin Predoi, Marcela Popa, Krzysztof Rokosz, Steinar Raaen, Ioana Cristina Marinas

**Affiliations:** 1 National Institute of Materials Physics, Atomistilor Street, No. 405A, 077125 Magurele, Romania; ciobanucs@gmail.com (S.C.C.); simonaiconaru@gmail.com (S.L.I.); 2 Life, Environmental and Earth Sciences Division, Research Institute of the University of Bucharest (ICUB), University of Bucharest, 060023 Bucharest, Romania; carmen.chifiriuc@gmail.com; 3 Department of Microbiology, Faculty of Biology, University of Bucharest, 1-3 Aleea Portocalelor Str., District 5, 060101 Bucharest, Romania; marcela.popa@bio.unibuc.ro (M.P.); ioana.cristina.marinas@gmail.com (I.C.M.); 4 Biological Sciences Division, The Romanian Academy, 25, Calea Victoriei, 010071 Bucharest, Romania; 5 Department of Mechanics, University Politehnica of Bucharest, BN 002, 313 Splaiul Independentei, Sector 6, 060042 Bucharest, Romania; predoi@gmail.com; 6 Faculty of Electronics and Computer Science, Koszalin University of Technology, Sniadeckich 2, PL 75-453 Koszalin, Poland; rokosz@tu.koszalin.pl; 7 Department of Physics, Norwegian University of Science and Technology (NTNU), Realfagbygget E3-124 Høgskoleringen 5, NO 7491 Trondheim, Norway; steinar.raaen@ntnu.no

**Keywords:** sage, antimicrobial applications, zinc doped hydroxyapatite, morphology, dextran

## Abstract

In the present study, sage-coated zinc-doped hydroxyapatite was incorporated into a dextran matrix (7ZnHAp-SD), and its physico-chemical and antimicrobial activities were investigated. A 7ZnHAp-SD nanocomposite suspension was obtained using the co-precipitation method. The stability of the nanocomposite suspension was evaluated using ultrasound measurements. The stability parameter calculated relative to double-distilled water as a reference fluid highlights the very good stability of the 7ZnHAp-SD suspension. X-ray diffraction (XRD) experiments were performed to evaluate the characteristic diffraction peak of the hydroxyapatite phase. Valuable information regarding the morphology and chemical composition of 7ZnHAp-SD was obtained via scanning electron microscopy (SEM), energy-dispersive X-ray spectroscopy (EDS), and X-ray photoelectron spectroscopy (XPS) studies. Fourier-transform infrared spectroscopy (FTIR) measurements were performed on the 7ZnHAp-SD suspensions in order to evaluate the functional groups present in the sample. Preliminary studies on the antimicrobial activity of 7ZnHAp-SD suspensions against the standard strains of *Staphylococcus aureus* 25923 ATCC, *Enterococcus faecalis* 29212 ATCC, *Escherichia coli* 25922 ATCC, and *Pseudomonas aeruginosa* 27853 ATCC were conducted. More than that, preliminary studies on the biocompatibility of 7ZnHAp-SD were conducted using human cervical adenocarcinoma (HeLa) cells, and their results emphasized that the 7ZnHAp-SD sample did not exhibit a toxic effect and did not induce any noticeable changes in the morphological characteristics of HeLa cells. These preliminary results showed that these nanoparticles could be possible candidates for biomedical/antimicrobial applications.

## 1. Introduction

Nanotechnology is an emerging field of science that allows the obtaining of materials with controllable dimensions and properties. Thus, the application of nanomaterials in dentistry can lead to the prevention, diagnosis, and treatment of oral/dental diseases [1]. The nanobiomaterials used in dentistry are based on carbon, silica, polymers, hydroxyapatite, hydrogel, etc. [2]. Currently, the use of hydroxyapatite (HAp), which is a calcium phosphate-based compound, in implantology and dentistry is growing continuously [3]. HAp, due to its chemical composition that mimics the inorganic part of bone tissue and has good biocompatibility and bioactivity, allows for an improved osteointegration and longevity of the HAp-coated titanium implants [3,4,5]. The hydroxyapatite bioactivity and its ability to induce the biomineralization process suggest that it can be used to prevent dentine hypersensitivity after a bleaching procedure [6]. Also, in order to prevent caries, hydroxyapatite is added to the toothpaste, which helps reduce the demineralization process and sustain the formation of protective coatings on the teeth’s surface [7,8]. The hydroxyapatite obtained at the nanometric scale has the ability to prevent the development of dental plaque due to the fact that it can adsorb pathogenic bacteria in the mouth, proteins, and dextran in saliva [9]. These biomedical uses of hydroxyapatite nanopowders suggest that it may be the right candidate for application in preventive and aesthetic medicine.

It is well known that zinc ions (Zn) have an important role in regulating enzyme activities and promoting bone mineralization and formation [10]. Previous studies have shown that materials based on hydroxyapatite doped with zinc have good antimicrobial activity against bacteria found in the oral cavity [11]. The results of the studies conducted by Takatsuka, T. et al. [12] revealed that zinc ions have the ability to inhibit dentine demineralization. Also, *in vitro* tests showed that the presence of zinc in biomaterials inhibits the formation of cariogenic biofilm and promotes the preservation of collagen. On the other hand, zinc deficiency can lead to more cavities, especially in the molars [13]. Therefore, these effects of zinc make it suitable to be used as a therapeutic agent [14]. Dextran-coated hydroxyapatite obtained by Liljemark, W.F. et al. and Rolla, G. et al. showed a strong affinity to *S. mutans* compared to the uncoated hydroxyapatite [15,16].

Since ancient times, essential oils have been used in order to ameliorate oral pain or mouth odor, and recently, they have been used in root canals and in periodontal treatments [17,18]. Sage (*Salvia officinalis, S. officinalis*) is a plant cultivated worldwide and is used for food seasoning and flavoring [19]. There are some studies that highlight the antibacterial, antifungal, anti-inflammatory, antitumor, and antioxidant activities of *S. officinalis* [20,21]. The enhanced antimicrobial activity against both clinical and reference microbial strains that are found in the oral cavities of *Salvia officinalis* was underlined in the studies conducted by De Oliveira, J.R. and coworkers [20]. Furthermore, no cytotoxic effect on the murine macrophage (RAW 264.7) cell line was observed. Therefore, the ability of *Salvia officinalis* to prevent the development of pathogenic bacteria without affecting the surrounding tissue makes it suitable for use as a therapeutic agent [20]. In this context, the fabrication of a new composite material based on sage-coated magnesium-doped hydroxyapatite in a dextran matrix could provide a new antimicrobial agent that could be used as an active ingredient in oral hygiene products, dental treatments, or other relevant antimicrobial applications.

In the present study, both physico-chemical and antimicrobial studies were performed on sage-coated zinc-doped hydroxyapatite in a dextran matrix (7ZnHAp-SD). The stability of sage-coated zinc-doped hydroxyapatite in a dextran matrix (7ZnHAp-SD) was analyzed by performing ultrasound studies (US). Furthermore, the scanning electron microscopy method was used in order to obtain information on the 7ZnHAp-SD morphology. The chemical composition of the obtained suspension was studied using energy-dispersive X-ray (EDX) and X-ray photoelectron spectroscopy (XPS). The structure of the 7ZnHAp-SD powder was examined using X-ray diffraction (XRD). The FTIR measurements were performed on the 7ZnHAp-SD sample in order to prove the presence of functional groups. The *in vitro* evaluation of the antimicrobial efficiency of 7ZnHAp-SD suspension was performed using the following standard strains: *Staphylococcus aureus* 25923 ATCC, *Enterococcus faecalis* 29212 ATCC, *Escherichia coli* 25922 ATCC, and *Pseudomonas aeruginosa* 27853 ATCC. Additionally, preliminary information about the biocompatibility of the 7ZnHAp-SD was obtained via the MTT assay using a human cervical adenocarcinoma (HeLa) cell line.

## 2. Materials and Methods

### 2.1. Materials

In order to synthesize sage-coated zinc-doped hydroxyapatite in a dextran matrix (7ZnHAp-SD, Ca_10−x_Zn_x_(PO_4_)_6_(OH)_2_; x_Zn_ = 0.07) using the co-precipitation technique, various reagents (calcium nitrate tetrahydrate, diammonium hydrogen phosphate, and zinc nitrate hexahydrate) with higher purity (≥99.0%) were purchased from Sigma Aldrich, St. Louis, MO, USA. Ammonium hydroxide, dextran, H(C_6_H_10_O_5_)_n_ (Sigma Aldrich, St. Louis, MO, USA, Mr~40,000), sage (*Salvia officinalis*) essential oil, ethanol absolute, C_2_H_6_O (Sigma Aldrich, St. Louis, MO, USA, purity ≥ 99.8%), and double-distilled water were also purchased from Sigma Aldrich.

### 2.2. Synthesis of Sage-Coated Zinc-Doped Hydroxyapatite in Dextran

The sage-coated zinc-doped hydroxyapatite in a dextran matrix (7ZnHAp-SD) suspension was obtained using an adapted co-precipitation technique [22,23]. The synthesis of Ca_10−x_Zn_x_ (PO_4_)_6_(OH)_2_ with x_Zn_ = 0.07 was effectuated at room temperature (RT). The (Ca + Zn)/P molar ratio was set to 1.67 in agreement with the previous studies [22,23]. After dissolving Ca(NO_3_)_2_·4H_2_O and Zn(NO_3_)_2_·6H_2_O in a beaker, a solution-based Zn and Ca was obtained. This solution was added drop-by-drop to a solution of (NH_4_)_2_·HPO_4_ and sage-dextran (1 mL of *Salvia officinalis* essential oil per 100 mL of dextran (10%)). The value of the pH solution was maintained at 10 by adding NH_4_OH during the synthesis process. After four hours of stirring, the solution was centrifuged and redispersed in a sage–dextran solution (1 mL of *Salvia officinalis* essential oil per 100 mL of dextran 10%). This process was repeated 5 times. The resulting precipitate was redispersed in a dextran–sage solution and stirred for 24 h at 40 °C.

### 2.3. Characterization Methods

The ultrasound measurements were conducted in order to evaluate the stability of sage-coated zinc-doped hydroxyapatite in a dextran matrix (7ZnHAp-SD) as the final concentrate suspension [22,24]. For this experiment, the 7ZnHAp-SD suspension was stirred at 900 rot/min for 20 min in a special cubic vessel according to the previous studies [22,24]. The vessel had coaxially opposed ultrasonic transducers, each with a central frequency of 25 MHz. Immediately after stopping the continuous stirring, the ultrasonic signals transmitted from one transducer to the other were recorded. These ultrasonic signals were transmitted every 5 s. The total duration was 5000 s. The structure of the 7ZnHAp-SD powder was examined via X-ray diffraction (XRD). The powder was analyzed using a Bruker D8 Advance diffractometer with CuKα (λ = 1.5418 Å) radiation (Bruker, Karlsruhe, Germany) equipped with a high-efficiency LynxEye™ 1D linear detector. The patterns were obtained in the 2θ range of 20–60°. The step size was 0.02° and the dwell time was 5 s. To study the morphology of the 7ZnHAp-SD particles in suspension, a scanning electron microscope (FEI Quanta Inspect F, Hillsboro, OR, USA) equipped with an energy-dispersive X-ray (EDX) attachment was used. From the obtained SEM micrographs, the particle size distribution was obtained. The particle size distribution was obtained after counting over 700 nanoparticles of 7ZnHAp-SD using the ImageJ software (Image J 1.51j8, National Institutes of Health, Bethesda, MD, USA) [25]. The experimental histogram containing the obtained values was fitted to a Gaussian function. The FTIR measurements were performed using the 7ZnHAp-SD sample in order to confirm the presence of functional groups. The FTIR measurements were made using a Spectrum BX Spectrometer. Therefore, 1% of the 7ZnHAp-SD powder was mixed and ground well with 99% dried KBr. The powder mixture obtained was pressed for 2 min with a load of 5 tons. The tablets obtained were used for FTIR measurements. The FTIR spectrum was recorded in the spectral range of 450 to 2000 cm^−1^. The FTIR parameters used were previously described in detail [26]. The 7ZnHAp-SD sample was also studied via XPS analysis. The XPS spectra were acquired with the aid of an SES 2002 instrument (Scienta Omicron, Taunusstein, Germany), using a monochromatic Al K(alpha) (hν = 1486.6 eV) X-ray source (Scienta Omicron, 18.7 mA, 13.02 kV). Herein, the XPS experiments were performed according to the procedure described in detail elsewhere [27]. The XPS experimental data obtained were analyzed using the Casa XPS 2.3.14 software (Shirley background type). For the XPS experiments, all the binding energy (BE) values were charge-corrected to the C 1 s line at 284.8 eV.

### 2.4. Antimicrobial Assay

Evaluation of the compounds’ antimicrobial activity was performed using the following standard strains: *Staphylococcus aureus* 25923 ATCC, *Enterococcus faecalis* 29212 ATCC, *Escherichia coli* 25922 ATCC, and *Pseudomonas aeruginosa* 27853 ATCC. The antimicrobial activity was tested using the Kirby–Bauer modified method. For this purpose, 0.5 McFarland suspension was inoculated on Mueller Hinton agar and 10 µL of 10 mg/mL testing substance was spotted. After 24, 48, and 72 h of incubation, the diameter of the zone of inhibition (DZI) was measured using a ruler. Broth dilution testing of the antimicrobial activity was performed in 96-well plates. Serial binary dilutions of the test compounds (reaching concentrations between 5 and 0.009 mg/mL) were performed, and 10^6^ CFU/mL (colony-forming units) microbial suspension was added and allowed to come into contact for 24 h at 37 °C. After incubation, due to the opacity of the culture medium, 5 µL from each well was seeded on agar medium to determine the minimum inhibitory concentration (MIC). Antibiofilm assay was performed in the same manner as MIC determination; shortly after incubation, the 96-well plates were discarded, washed, fixed with alcohol, stained with crystal violet, and the bound dye was removed from the stained cells using acetic acid. Biofilm formation was quantified by measuring the optical density of the suspension at 490 nm.

### 2.5. Cytotoxicity Assay

The toxicity of the 7ZnHAp-SD sample was evaluated using human cervical adenocarcinoma (HeLa) cells. For this purpose, the cells were trypsinized, counted, and seeded in well plates of 96-well plates with a density of 7.5 × 10^3^ cells per well. Afterward, the plates were incubated in Dulbecco’s modified medium (DMEM) at a temperature of 37 °C previously enriched with heat-inactivated fetal bovine serum in an atmosphere containing a CO_2_ concentration of 5%. In order to evaluate the cytotoxicity of the samples, the cells were seeded in Petri dishes, incubated with 15.625 µg/mL of 7ZnHAp-SD, and kept at a temperature of 37 °C in an atmosphere with 5% CO_2_ for 24, 48, and 72 h. The cell viability was assessed using an MTT colorimetric assay. For this purpose, the cells were incubated for 4 h at a temperature of 37 °C in a humid atmosphere containing 5% CO_2_ with 0.1 mg/mL of MTT solution [3-(4,5dimethylthiazolyl)-2,5-diphenyltetrazolium bromide]. The cell viability was quantified based on the optical density of the medium recorded at 595 nm using a TECAN spectrophotometer. The percentage of viable cells was determined by comparison with a control sample, which was considered to have a viability of 100% (culture medium without 7ZnHAp-SD). Furthermore, the morphological features of the HeLa cells incubated with the 7ZnHAp-SD sample were also evaluated using fluorescence microscopy with an Olympus IX71 microscope (Olympus, Tokyo, Japan).

### 2.6. Statistical Analysis

Data were expressed as mean ± SD, and statistical analysis was performed using GraphPad Prism v10. Data were analyzed using ordinary one-way ANOVA using Tukey’s multiple comparisons test, with a single pooled variance for multiple comparisons between different time intervals of incubation. The significance level was set at *p* < 0.05.

## 3. Results

Creating a biomaterial with antimicrobial properties that could be used as a biofilm in dentistry and could lead to the prevention of caries, periodontitis, and other dental diseases is a challenge at this moment. Taking into account the fact that composites based on hydroxyapatite are essential in the field of bone tissue engineering, in this study, we focused on the development of a stable suspension-based *Salvia officinalis*-coated zinc-doped hydroxyapatite in a dextran matrix that could be successfully used in various dental applications, such as dental implants and dental prostheses, and even in the periodontal field.

The stability of suspensions and the size of particles at the nanometric scale play an important role due to their ultra-small size, high surface-to-volume ratio, and special physico-chemical properties. As a result, in this study, we analyzed the stability of 7ZnHAp-SD suspensions in a concentrated form using ultrasonic measurements. The stability of the suspension was evaluated before the assessment of the antimicrobial activity of the material (approximately five weeks after it was obtained following the synthesis process). For a correct evaluation of the stability of the 7ZnHAp-SD suspension, the fluid with the best stability (double-distilled water) was used as a reference.

**Figure 1:**
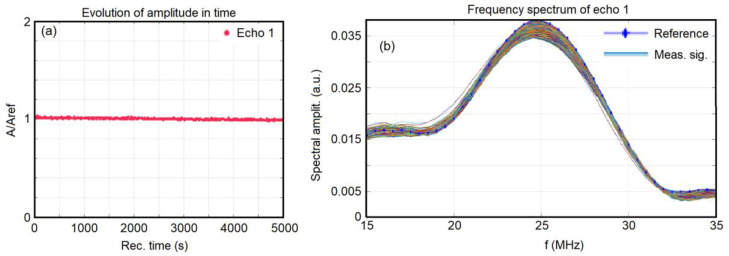
Evolution in time of the ultrasonic signal relative amplitudes through the 7ZnHAp-SD suspension (**a**) and the frequency spectra of all recorded signals and the reference liquid and the 7ZnHAp-SD suspension (**b**).

An important role in evaluating the stability of the 7ZnHAp-SD solution is played by the amplitudes of the transmitted signals. These amplitudes of the transmitted signals were determined relative to the double-distilled water (taken as the reference liquid), and the measurements were performed under identical conditions. The relative amplitudes obtained for the 7ZnHAp-SD suspension are presented in Figure 1a. It can be seen that the relative amplitudes diminish very slowly during the testing period. Figure 1b shows the ultrasonic signals transmitted through the sample and transformed into their frequency spectra, superimposed for all 1000 recorded signals. It was observed that a general tendency was represented by the fact that the maximum of all the amplitudes was at the central frequency of the transducers (25 MHz).

**Figure 2:**
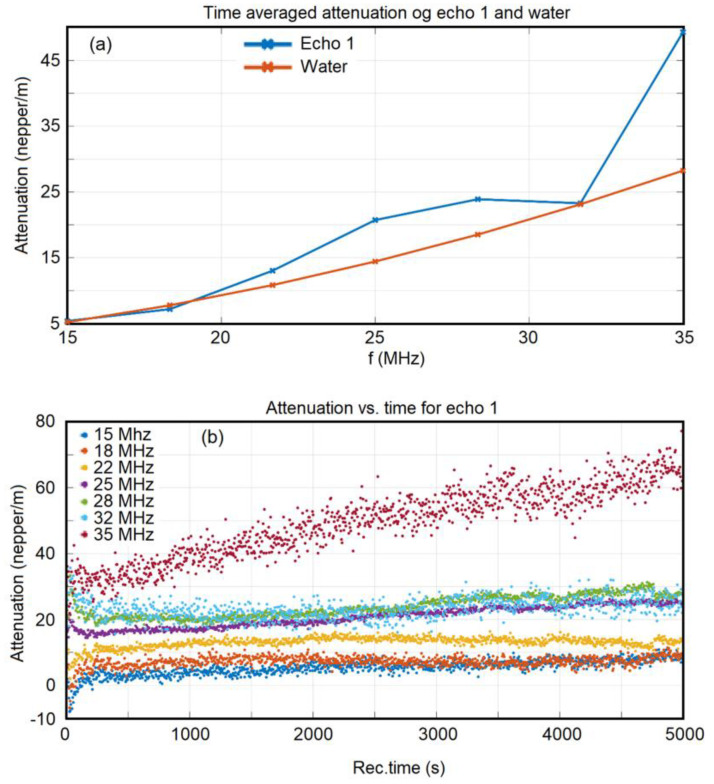
Attenuation of ultrasonic signals vs. frequency for (**a**) and attenuation of ultrasonic signals vs. time for 7ZnHAp-SD suspension (**b**).

Moreover, the evolution of t**he** spectral amplitudes was extremely slow and very close to the pattern of the reference liquid. These spectra were averaged over 1000 time records, and the attenuation of signals could thus be obtained (Figure 2a). The attenuation in this sample was very close to the attenuation in the reference liquid, with the noteworthy exception of the attenuation at 35 MHz. At this highest frequency, the nanoparticle suspension strongly interacted with the ultrasonic signal, absorbing a larger ratio of the incident energy. The averaged stability parameter, $S=\frac{\bar{dA}}{Adt}$ calculated from the slope of the interpolation line in Figure 1a, was S = 3.46 × 10^−6^ (1/s) after the initial 300 s. This value of the averaged stability parameter represents very good stability compared to that of the double-distilled water. In general, the attenuation at each frequency remained constant, and most of the constants were positive. The only exception was for 15–18 MHz, for which the attenuation was small but negative, which was influenced by the 7ZnHAp-SD nanoparticles in suspension. It was relevant to present the evolution of attenuation in the sample for several frequencies during the duration of the experiment (Figure 2b). In general, the attenuation increased with time. Only the 22 MHz component slowly decreased. Also, the 35 MHz component exhibited a strong increase in time, which almost doubled the attenuation from 30 dB/m to 65 dB/m during the sedimentation of the suspension. During the initial 100 s of the experiment, the larger nanoparticles settled first, accompanied by an increase in attenuation for the lower frequencies (15, 18, and 22 MHz) in the spectrum and a decrease in attenuation for the higher frequencies (25, 28, 32, and 35 MHz). The lowest attenuations can be explained by the presence of larger 7ZnHAp-SD nanoparticles in the suspension, which then settled down and the smaller nanoparticle absorbed a higher amount of ultrasonic wave energy.

**Figure 3:**
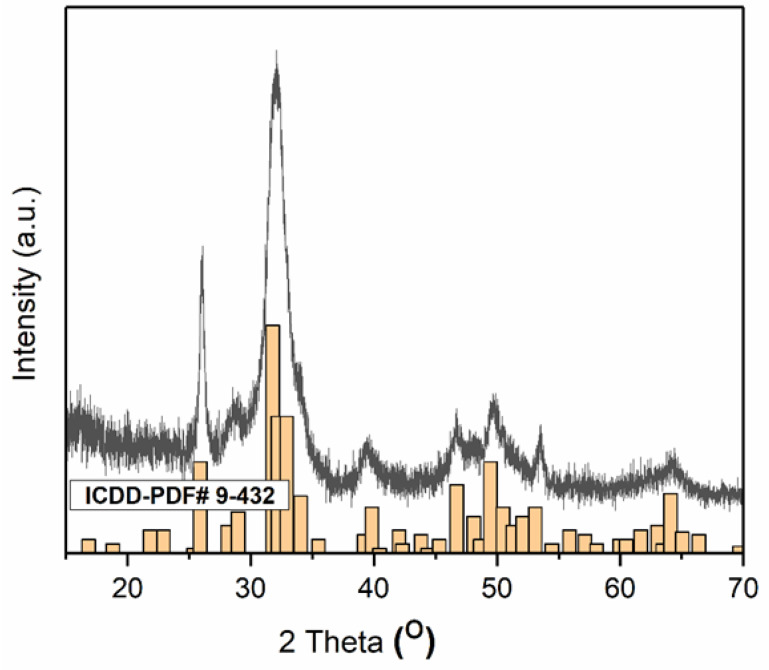
XRD patterns of 7ZnHAp-SD and the lines of the ICDD-PDF#9-432 reference file for hexagonal hydroxyapatite (in orange).

The XRD patterns of the 7ZnHAp-SD powders and the lines of the ICDD-PDF#9-432 reference file for hexagonal hydroxyapatite (in orange) are shown in Figure 3. The XRD patterns collected for the 7ZnHAp-SD powders revealed broad diffraction maxima. The diffraction peaks of the 7ZnHAp-SD sample corresponded to the reference lines associated with hexagonal hydroxyapatite (ICDD-PDF#9-432). The assessment of lattice parameters and crystal size obtained from Rietveld refinement calculations [28] showed that the a-lattice parameter was equal to 9.46 Å, whereas the c-lattice parameter was equal to 6.90 Å. The estimated crystal size was 17 ± 1 nm.

**Figure 4:**
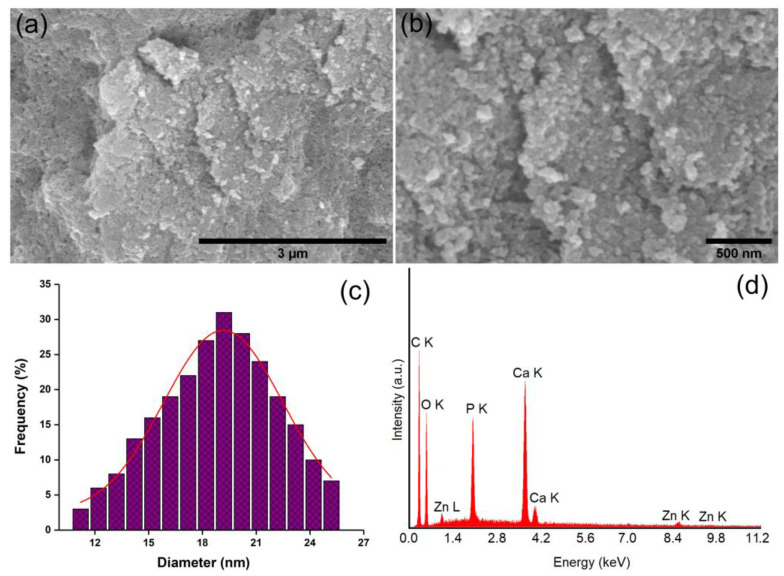
Low (**a**) and high (**b**) magnification micrographs of 7ZnHAp-SD particles in suspension; size distribution (**c**) and EDS spectrum (**d**) of the 7ZnHAp-SD suspension.

The size and distribution of the 7ZnHAp-SD particles in the suspension were determined via SEM analysis. Figure 4a,b shows low- and high-magnification micrographs of 7ZnHAp-SD particles in the suspension. The particles had a nanometric size and a spherical shape. The agglomeration of the 7ZnHAp-SD nanoparticles was due to the analysis method.Drying under vacuum at room temperature the drop of 7ZnHAp-SD suspension on the carbon strip, may led to the agglomeration of the nanoparticles. The size distribution obtained after counting over 700 nanoparticles of 7ZnHAp-SD is presented in Figure 4c. The calculated average size was 19.2 ± 2 nm. The EDS spectrum of the 7ZnHAp-SD particles is also shown in Figure 4d. The presence of the main constituent elements such as calcium, phosphorus, oxygen, zinc, and carbon is highlighted. It can be clearly seen that no additional lines specific to other elements were detected in the EDS spectrum of 7ZnHAp-SD.

**Figure 5:**
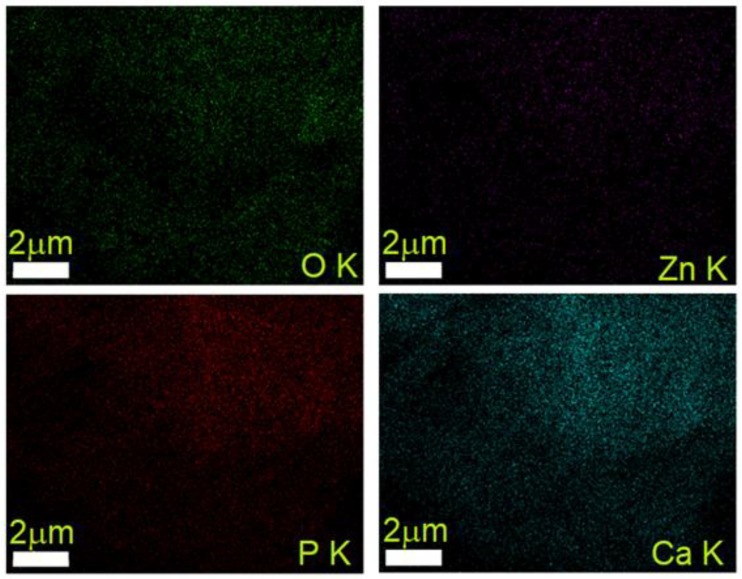
Elemental EDS mapping analysis of the 7ZnHAp-SD suspension.

The EDS mapping analysis of 7ZnHAp-SD revealed a uniform distribution of the constituent elements: Ca, P, Zn, and O (Figure 5). The C element was not presented in the EDS mapping because it came from both the sample and the carbon strip on which the drop of 7ZnHAp-SD suspension was deposited.

**Figure 6:**
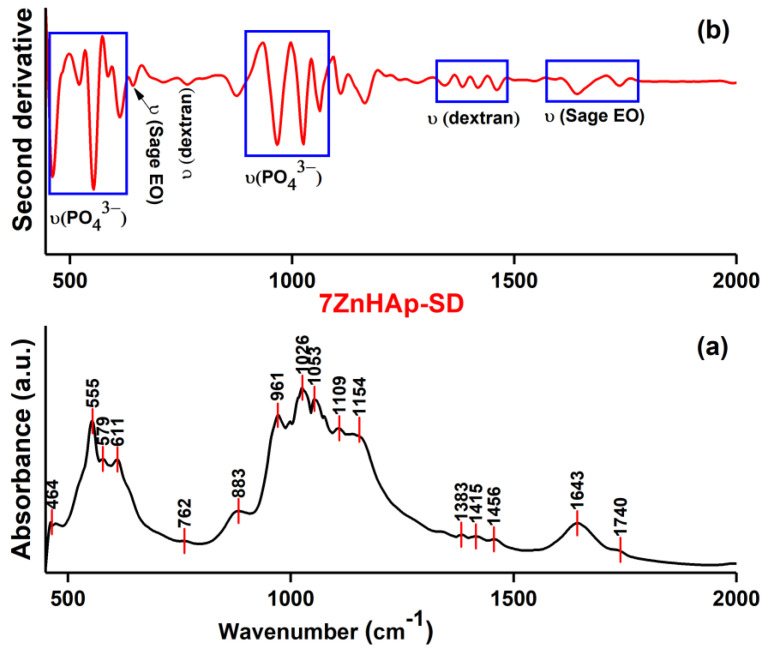
Absorbance (**a**) and second-derivative (**b**) FTIR spectra of the 7ZnHAp-SD sample.

Figure 6 depicts the typical absorbance and second-derivative FTIR spectra obtained for the 7ZnHAp-SD sample. The FTIR spectra (Figure 6a) show the presence of maxima that are specific to the hydroxyapatite, dextran, and sage essential oil structure. The maxima found at 464, 555, 579, and 611 cm^−1^ belong to the (ν_2_ and ν_4_) phosphate functional groups from the HAp structure [26]. Moreover, the maxima observed at about 961 cm^−1^ corresponded to the ν_1_ vibration mode of HAp phosphate functional groups [26]. At around 1026 and 1053 cm^−1^, maxima appeared due to the ν_3_ vibration of the phosphate groups. The typical maxima associated with the vibration of functional groups from the dextran structure were usually noticed in the 1500–750 cm^−1^ spectral region [29]. The presence of maxima in these regions was due to the vibration of C-O, CH_2,_ or C-OH functional groups from the dextran structure [29].

Also, in the FTIR spectra of the 7ZnHAp-SD sample, the maxima that appeared due to the presence of sage essential oil were observed. According to the results presented by Tulukcu, E. and collaborators [30], the sage essential oil fingerprint domain was found between 600 and 900 cm^−1^. The maxima that were noticed in this domain can be attributed, mainly, to the vibrations of nucleotides and aromatic amino acids [30]. Additionally, the shoulder observed at around 1740 cm^−1^ was typical for the carbonyl absorption of the triglyceride ester linkage [31]. Furthermore, the presence of pinene underlined by the maxima was noticed at about 1643 cm^−1^ in the FTIR spectra [31]. At the same time, the overlapping of the specific FTIR maxima of doped hydroxyapatite in the polymer matrix with that of the sage essential oil led to the appearance of broad maxima [32].

Figure 6b reveals the FTIR second-derivative curve obtained for the 7ZnHAp-SD sample. The second-derivative curve was obtained for the spectral domain between 450 and 2000 cm^−1^. The second-derivative profile provided more detailed information regarding the molecular structure and the presence of a specific band in the 7ZnHAp-SD sample. Therefore, these results confirmed the presence of molecular vibrations associated with the hydroxyapatite (PO_4_^3−^ bands), dextran (C-O, CH_2,_ and C-OH bands), and sage essential oil in the 7ZnHAp-SD sample [26,29,30,31,32].

**Figure 7:**
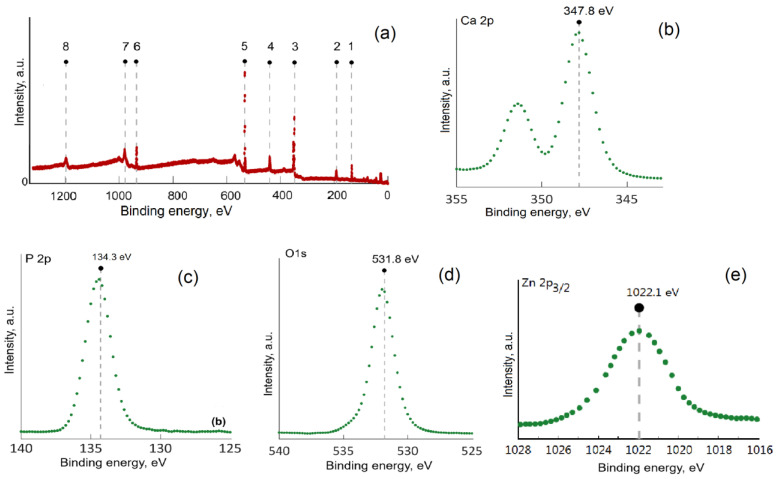
XPS general spectra of the 7ZnHAp-SD composite (**a**); and high-resolution spectra of calcium (**b**), phosphorus (**c**), oxygen, (**d**), and zinc (**e**).

In order to obtain additional information regarding the formation of sage-coated zinc-doped hydroxyapatite incorporated in a dextran matrix (7ZnHAp-SD), XPS studies were carried out. C1s (284.8 eV) was used to calibrate the binding energies. In Figure 7, both the general spectrum of 7ZnHAp-SD and the high-resolution XPS spectra for calcium (Ca 2p), phosphorus (P 2p), oxygen (O1s), and zinc (Zn2p3/2) are presented. As can be seen in Figure 7a, the general XPS spectrum reveals the presence of the Ca, P, O, Zn, and C elements, which proves the successful formation of 7ZnHAp-SD. Moreover, no additional peaks were observed in the general XPS spectrum, which shows that there are no impurities in the obtained sample. These results are in agreement with the data obtained from the X-ray diffraction and EDS studies. The high-resolution XPS spectrum of Ca 2p (Figure 7b) revealed that the maximum value was associated with the binding energy of 347.8 eV, which corresponded to Ca-O bonds in hydroxyapatite [33]. Figure 7c presents the high-resolution XPS spectra of P 2p localized at 134.3 eV and associated with P-O binding [33,34]. The high-resolution XPS spectra of O1 (Figure 7d) revealed a peak located at 531.1 eV corresponding to the P-O bonds [34,35]. In the high-resolution XPS spectrum of Zn 2p3/2 (Figure 7e), the maximum was identified at a BE of approximately 1022.1 eV, which highlights the fact that the valence of zinc did not change following the replacement of Ca^2+^ ions with Zn^2+^ [36,37,38]. The results of this study show that the sage-coated zinc-doped hydroxyapatite incorporated in a dextran matrix includes Zn ions in accordance with EDS studies [39].

For the *in vitro* evaluation of the antimicrobial activity of 7ZnHAp-SD suspensions, the following standard strains were used: *Staphylococcus aureus* 25923 ATCC, *Enterococcus faecalis* 29212 ATCC, *Escherichia coli* 25922 ATCC, and *Pseudomonas aeruginosa* 27853 ATCC.

The evaluation of the antimicrobial activity of the 7ZnHAp-SD compounds on agar showed that for the tested concentrations, the substances were active only on the *E. coli* bacterial strain. The values of the inhibition zones of the tested compounds (expressed in mm) are listed in Table 1.

The tested 7ZnHAp-SD samples that did not exhibit any antimicrobial activity on agar were further tested in nutrient broth at a concentration of 5 mg/mL. The results obtained for the minimum inhibitory concentrations (expressed in mg/mL) of 7ZnHAp-SD are presented in Table 2.

Bacterial adherence to inert substrates in order to form biofilms was inhibited by concentrations similar to those that inhibited bacterial growth. The results are shown in Table 3.

**Figure 8:**
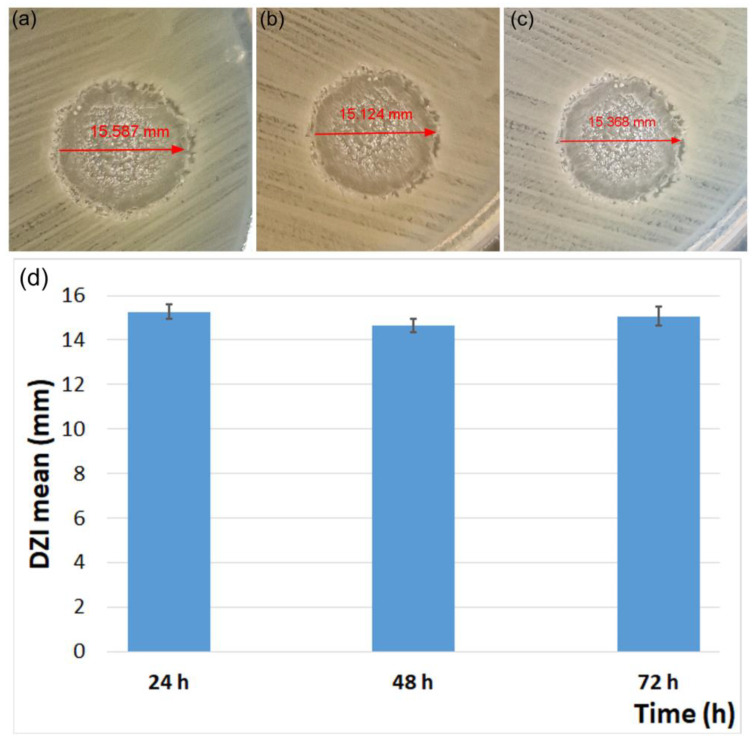
The diameters of the inhibitory zones of 7ZnHAp-SD nanoparticles obtained after 24 h (**a**), 48 h (**b**), and 72 h (**c**) against *E. coli* and the graphical representation of the values obtained for the DZI evaluated at various time intervals after incubation (**d**) (comparisons across time intervals were not statistically significant, *p* > 0.05 for up to 72 h) in the case of 7ZnHAp-SD nanoparticles against *E. coli*. Statistical analysis revealed that the changes in diameter at different time intervals are not statistically significant (*p* > 0.05).

The results presented in Table 2 highlight that the minimum inhibitory concentrations (expressed in mg/mL) for the 7ZnHAp-SD suspension activity against *S. aureus*, *E. faecalis*, *E. coli*, and *P. aeruginosa* were higher than 5 mg/mL. These results are in good agreement with previously reported data on the antimicrobial activity of sage essential oil-based materials, which showed antimicrobial properties at higher concentrations [20,40,41,42]. Miladinović et al. [40] in their study on the “antimicrobial activity of essential oil of sage from SERBIA” reported that the samples with higher concentration (2%) showed significantly better antimicrobial activity against *Bacillus subtilis* S, *Staphylococcus aureus* 6538, *Escherichia coli* 95, *Salmonella enteritidis*, and *Aspergillus niger* compared with the lower sample concentration (1%). Furthermore, Yıldırım et al. [41] in their study on the “Comparison of antioxidant and antimicrobial activities of Tilia (Tilia Argentea Desf Ex DC), Sage (*Salvia triloba* L.), and Black Tea (*Camellia sinensis*) extracts” demonstrated that for each 100 µg sample of the indicated extract, no activity was found on any of the tested microorganisms (*S. aureus* ATCC 25923, *C. albicans* ATCC 60193, *E. coli* ATCC 25922, *B. subtilis* ATCC 6633, and *P. aeruginosa* ATCC 10145). On the other hand, despite the absence of antimicrobial activity, Yıldırım et al. [41] reported that, the extracts of tea, sage, and tilia presented a high antioxidant activity. On the other hand, de Oliveira et al. [20], in their study on the “antimicrobial activity of noncytotoxic concentrations of *Salvia officinalis* extract against bacterial and fungal species from the oral cavity”, investigated the antimicrobial activity of *S. officinalis* extract in both reference strains (*S. aureus* ATCC 6538, *S. epidermidis* ATCC 12228, *S. mutans* ATCC 35688, *C. albicans* ATCC 18804, *C. tropicalis* ATCC 13803, and *C. glabrata* ATCC 90030) as well as in nine clinical isolates collected from the oral cavity of tuberculosis patients, totaling a number of 60 microbial strains. Their study demonstrated that all the tested microbial strains were eliminated when using an *S. officinalis* extract of 50.0 mg/mL. Moreover, their results emphasized that for lower concentrations of 25.0 mg/mL and 12.5 mg/mL, the microorganisms were only partially eliminated. In addition, their studies also highlighted that for concentrations below 12.5 mg/mL, there was no antimicrobial effect for any of the tested microorganisms [32]. In addition, the results reported by de Oliveira et al. [20] demonstrated that the tested *S. epidermidis* strains showed the most susceptibility to *S. officinalis* extract and that among all the tested microorganisms, the *S. mutans* bacterial strains were the most resistant to *S. officinalis* extract, even though the 10 isolates analyzed were completely eliminated when exposed to the extract at a concentration of 50.0 mg/mL. Also, data on the fungicidal activity of *Salvia officinalis* extracts on *Candida* spp were reported. Sookto et al. [43] reported the antifungal activity of *S. officinalis* essential oil on the reference strain *C. albicans* 90,028 and two fungal clinical isolates. Furthermore, in order to obtain additional information regarding the antimicrobial activity of the 7ZnHAp-SD nanoparticles against *E. coli* at different time intervals, the diameters of the inhibitory zones were measured. The results are presented in Figure 8a–d. The statistical analysis revealed that the changes in diameters for the three different time intervals tested were not statistically significant (*p* > 0.05). The images representing the DZI are depicted in Figure 8a–c, and the values for the obtained DZI are graphically represented as the mean ± SD in Figure 8d. These results are in agreement with the results presented by Han et al. [44] and, as further shown in their studies, the diameter of the inhibition zone did not change with the incubation time because in the diffusimetric method, the active principle diffuses in the medium proportional to the active principles’ concentration, which does not change over time. The images of the experiment are shown in Figure 8a–c.

Even though the mechanisms of antimicrobial activity of the essential oil and of various composites based on essential oils and metal ions that are known to possess antimicrobial activity are still unclear, taking into consideration the emergence and increase in antimicrobial-resistant microorganisms, the search for alternative methods involving materials science and plant products could be a beneficial approach and could lead to the development of novel strategies for antimicrobial agents. The preliminary results obtained in this study highlight the potential for developing new therapeutic agents based on sage-coated zinc-doped hydroxyapatite in a dextran matrix. Since *S. officinalis* essential oil has already been proven to exhibit strong *in vitro* antimicrobial properties [19,45,46,47,48], the next step is to design a more complex research approach to determine the correct dosage and parameters for the incorporation of *S. officinalis* in various biomaterials for medical and dental applications.

**Figure 9:**
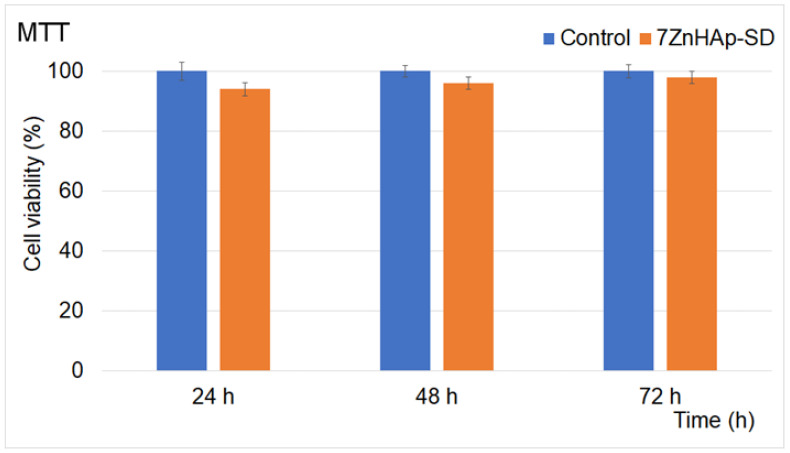
Graphical representation of the values obtained for HeLa cell viability after 24, 48, and 72 h of incubation in the presence of 7ZnHAp-SD. The results of the experiments are graphically represented as the mean ± SD. The data were statistically analyzed using paired and two-sample *t*-tests for means, with *p* ≤ 0.05 accepted as statistically significant.

The 7ZnHAp-SD nanoparticles were incubated with HeLa cells for 24, 48, and 72 h. Their cytotoxic activity was determined *in vitro* using the MTT assay. The data were graphically represented as the mean value ± SD of three independent experiments. A graphical representation of the MTT assay results is depicted in Figure 9.

The results of the cytotoxic activity of 7ZnHAp-SD determined from the MTT assay emphasized that after treatment for 24, 48, and 72 h, cell mortality was insignificant compared to the untreated cell culture used as a control. The graphical representation of the data obtained for the cell viability depicted in Figure 9 shows that the HeLa cell viability had a value of 94% relative to the control HeLa cells, which was considered to have 100% viability in the early 24 h stage of incubation. These findings indicated that the 7ZnHAp-SD nanoparticles exhibited good biological properties in the tested cell cultures. Furthermore, the data indicated that the cell viability of the cells treated with the 7ZnHAp-SD nanoparticles increased with an increase in the contact time of up to 96% after 48 h and 98% after 72 h. In addition, the results highlighted that the presence of dextran and sage essential oil in the composite materials did not confer toxicity against HeLa cells. The preliminary findings gathered from the MTT cytotoxic assays were in good agreement with previous studies that reported data on the biological properties of hydroxyapatite and hydroxyapatite-based composites [39,49,50,51,52]. In their previously reported studies on the “Biological response of human gingival fibroblasts to zinc-doped hydroxyapatite designed for dental applications—an *in vitro* study”, Badea et al. [53] highlighted that the decrease observed in cell viability was influenced by the concentration of the nanoparticles. Their findings suggested that the lowest number of cell viability was obtained after the cells were exposed to 500 μg/mL of ZnHAp and 62.5 μg/mL for HAp NPs. The results presented in their study indicated that the cytotoxicity of tested HAp and ZnHAp samples was influenced by their concentration. The results showed that in the case of low doses (lower than 15.625 μg/mL), there was no sign of alterations in cell morphology, any significant decrease in the cell viability, or disruption of the membrane integrity. Also, their findings suggested that low concentrations did not induce any modification in cell adhesion, but it influenced cell proliferation. On the other hand, their results concluded that exposure to higher doses (12 μg/mL) led to a decrease in the cell proliferation rate and the inhibition of cell adhesion. Moreover, their study revealed that exposure to higher doses of HAp and ZnHAp affected the F-actin cytoskeleton filaments but did not induce an inflammatory response or a cell membrane leakage. Furthermore, Sonmez et al. [54], in their study on “Toxicity assessment of hydroxyapatite nanoparticles in rat liver cell model *in vitro*”, reported that the exposure to a high concentration of hydroxyapatite nanoparticles caused both genotoxicity and cytotoxicity in hepatocytes because of the oxidative stress. The results on the toxicity of Hap and the level of toxicity indicated that they were caused by the shape of the nanoparticles, and the cell type that was investigated was also reported in other studies [55,56].

**Figure 10:**
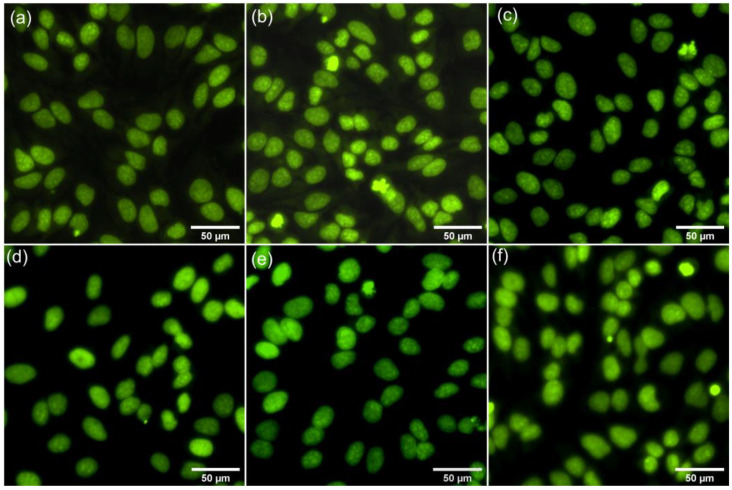
Two-dimensional fluorescence microscopy images of the morphology of HeLa cells treated with 7ZnHAp-SD for 24 h, 48 h, and 72 h (**d**–**f**) relative to the untreated Hela cell culture used as a control (**a**–**c**).

Furthermore, additional information on the effects of the 7ZnHAp-SD nanoparticles on the morphology of HeLa cells was also obtained using fluorescence microscopy. The two-dimensional fluorescence microscopy images of the untreated HeLa cells used as a control as well as those for the HeLa cells treated with 7ZnHAp-SD nanoparticles for 24 h, 48 h, and 72 h are illustrated in Figure 10a–f.

The micrographs depicted in Figure 10 highlighted that the morphological features of the HeLa cells treated with 7ZnHAp-SD nanoparticles were not altered even after 72 h of contact time compared to the morphology exhibited by the untreated Hela cell culture used as a control. These images clearly suggest that cells treated with 7ZnHAp-SD nanoparticles did not exhibit any observable alterations in their morphological characteristics. Moreover, the cells incubated with the 7ZnHAp-SD nanoparticles appeared to display a uniform and homogenous distribution with conventional cellular shapes typical of the HeLa cell line, presenting distinct edges and centrally placed nuclei. These results are in good agreement with the data obtained from the quantitative MTT studies and suggest that the 7ZnHAp-SD nanoparticles present good biocompatibility and that they could be considered for future biomedical applications.

**Figure 11:**
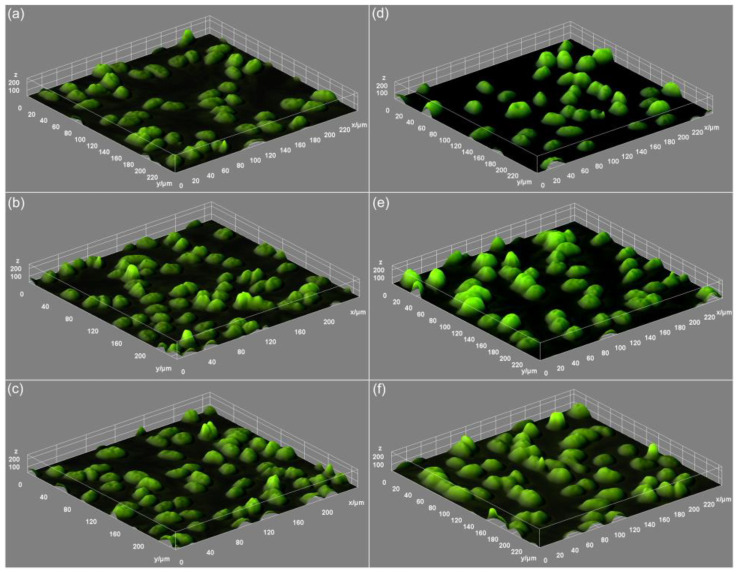
Three-dimensional representation of the fluorescence micrographs of the morphology of HeLa cells treated with 7ZnHAp-SD for 24 h, 48 h, and 72 h (**d**–**f**) compared to the untreated Hela cell culture used as a control (**a**–**c**).

Complementary information about the modification that could appear in the morphology of the HeLa cells treated with the 7ZnHAp-SD nanoparticles was obtained from the 3D representation of the fluorescence microscopy images (Figure 11).

The 3D images depicted in Figure 11 highlighted that there were no visible alterations of the HeLa cell morphology in the case of the cells treated with 7ZnHAp-SD nanoparticles for all the studied time intervals compared to the untreated cell culture used as a control.

## 4. Conclusions

The XRD analysis indicated that a one-phase material consisting of hydroxyapatite was obtained as a result of the coprecipitation method used. The stability of the 7ZnHAp-SD suspensions was very good compared to that of the double-distilled water. The SEM analysis of the 7ZnHAp-SD suspensions revealed the spherical shape of the particles. The EDS mapping analysis of 7ZnHAp-SD confirmed the homogeneous distribution of the main constituent elements. The FTIR studies confirmed the presence of molecular features typical of the hydroxyapatite, dextran, and sage essential oil structure in the studied powder. Overall, our preliminary findings on the cytotoxic properties of the 7ZnHAp-SD nanoparticles indicated that these composites presented no toxicity towards HeLa cells for the three tested incubation periods. Furthermore, microscopic observations also suggested that the 7ZnHAp-SD nanoparticles did not induce any noticeable modification of the morphological characteristics of the cells. The preliminary results obtained in this study highlighted the potential of developing new therapeutic agents based on sage-coated zinc-doped hydroxyapatite in a dextran matrix. The further step consists of performing more complex research studies in order to determine the correct dosage and parameters of the incorporation of *S. officinalis* in various biomaterials for biomedical and/or antimicrobial applications (such as dentistry and orthopedics).

## Figures and Tables

**Table 1:** Inhibition zones of the tested compounds (expressed in mm).

Compound	*S. aureus*	*E. faecalis*	*E. coli*	*P. aeruginosa*
7ZnHAp-SD	NA	NA	16	NA

NA—not active.

**Table 2:** Minimum inhibitory concentrations (expressed in mg/mL).

Compound	*S. aureus*	*E. faecalis*	*E. coli*	*P. aeruginosa*
7ZnHAp-SD	>5	>5	>5	>5

**Table 3:** Minimum concentrations that inhibited bacterial adherence to inert substrates (expressed in mg/mL).

Compound	*S. aureus*	*E. faecalis*	*E. coli*	*P. aeruginosa*
7ZnHAp-SD	>5	>5	>5	>5

## Data Availability

Data are available from the corresponding author upon reasonable request.
